# A Novel De Novo Chromosomal Insertion, 46 XX, ins(7:13)(p14; q14.2q21.1) is Related to the Embryo Development Arrest Following Assisted Reproductive Technique

**DOI:** 10.18502/jri.v21i4.4336

**Published:** 2020

**Authors:** Azam Azargoon, Nahid Azad

**Affiliations:** 1- Abnormal Uterine Bleeding Research Center, Semnan University of Medical Sciences, Semnan, Iran; 2- Department of Infertility, Amir-AL-Momenin Hospital, Semnan University of Medical Sciences, Semnan, Iran; 3- Department of Reproductive Biology, Semnan University of Medical Sciences, Semnan, Iran

**Keywords:** Assisted reproductive technique, Chromosomal rearrangement, Cytogenetic analysis, Infertility woman, IVF failure

## Abstract

**Background:**

Infertility is a problem affecting a large number of couples in the world. One of the causes of infertility can be chromosomal rearrangements such as insertions. In this case report study, the outcome of two intra-cytoplasmic sperm injection (ICSI) cycles of an infertile woman with de novo chromosomal insertion is explained.

**Case Presentation:**

A couple with a 10-year history of infertility referred to our infertility clinic. The husband had a daughter in his first previous marriage. The wife had a 7 and a 10 year history of infertility in the first and second marriages, respectively. In the first marriage, she reported a history of 2 failed intra-uterine insemination (IUI) cycles. In the second marriage, she had a history of 1 spontaneous abortion at 12 weeks of pregnancy, 4 failed IUI cycles, and 1 failed ICSI cycle. The couple was subjected to ICSI cycles twice and failed due to embryo development arrest. The couple referred for karyotyping. The husband showed a normal male karyotype. In comparison, the wife revealed an abnormal female karyotype with two rearrangements: chromosome 13 with an interstitial deletion between bands q14.2 and q21.1, and a derivative chromosome 7 containing this segment of chromosome 7 as an insertion onto short arm at the p14 position.

**Conclusion:**

To the best of our knowledge, this is the first report of insertion 46 XX, ins(7:13)(p14; q14.2q21.1) which is associated with the embryo development arrest following assisted reproductive technique.

## Introduction

Chromosomal rearrangement is a set of structural changes in chromosomes resulting in chromosomal abnormalities. It occurs when a double strand DNA breaks and rejoins aberrantly (1, 2). Insertions are rare chromosomal rearrangements with unidentified molecular mechanisms (3). People with infertility are prone to have chromosomal abnormality (4). It is estimated that 5.01% of infertile people have chromosomal abnormalities. The frequency of structural chromosomal abnormalities among infertile women was identified to be 1.87% using cytogenetic techniques (5).

In the present study, the outcome of two intra-cytoplasmic sperm injection (ICSI) cycles of an infertile woman with de novo chromosomal insertion, ins(7:13)(p14; q14.2q21.1) is reported.

## Case Presentation

A couple with a 10 year history of infertility referred to our infertility clinic. The man had a daughter of the first previous marriage. He was a 36-year-old man with no remarkable infertility factors. Also, he had normal semen analysis according to World Health Organization 2010 (WHO, 2012) with normal sperm parameters of 126×10^6^/*ml* concentration and 56% motility. The wife was a 33-year woman with a 7 and 10 year history of infertility in the first and second marriages, respectively. She had normal regular menstrual cycles beginning at 14 years. In the first marriage, she reported a history of 2 failed intra-uterine insemination (IUI) cycles. In the second marriage, she had a history of 1 spontaneous abortion at 12 weeks of pregnancy, 4 failed IUI cycles, and 1 failed ICSI cycles. Ovaries, uterine and fallopian tube of the woman showed normal appearance via sonographic evaluations. The hormonal examination also illustrated a normal range of LH, FSH, TSH, and AMH. In reply to their request, the couple underwent two ICSI cycles in Semnan infertility clinic with a 3-year interval (March 2016–May 2019).

Ovarian stimulation with recombinant FSH (Gonal-F, Merck Inc, Germany) was initiated on day 3 of the cycle at the dose of 150 *IU* daily. From stimulation day 6, transvaginal ultrasound was performed. The gonadotropin dose was adjusted according to serial ultrasound monitoring. The GnRH antagonist (Cetrorelix acetate) (Cetrotide; Serono International S.A., Switzerland) 0.25 *mg/S.C* was administered on a daily basis when a dominant follicle reached 13 *mm* in maximum diameter. When at least three follicles with a diameter of ≥17 *mm* were observed by ultrasound, final oocyte maturation was triggered with a single injection of triptorelin (Decapeptyl®; Ferring, Sweden) 0.2 *mg/S.C* because the patient was at the risk of developing ovarian hyperstimulation syndrome (OHSS).

Oocyte–cumulus complexes were aspirated transvaginally 35 *hr* following decapeptyl injection and placed in incubator (CO_2_ 6%, and O_2_ 5%) at 37*°C* for 4 *hr*. Afterwards, the oocytes were denuded enzymatically (Exposure to hyaluronidase) and mechanically. Metaphase II oocytes with no severe abnormality were selected for microinjection. Eppendorf micromanipulator mounted on an Olympus inverted microscope was applied for performing ICSI. Finally, injected oocytes were cultured for 5 days in global medium (lifeGlobal). The ICSI outcomes are demonstrated in table 1.

**Table 1.** Main outcomes of two ICSI cycles with a 3 year interval

**Cycle**	**Retrieved oocytes**	**GV oocytes**	**MI oocytes**	**MII oocytes**	**Fertilized oocytes**	**2-cell embryos**	**3–8 cell embryos**	**Morulla (72 *hr*)**	**Blastocyst (96 *hr*)**
**First**	14	-	3	11	10	10	8	-	-
**Second**	13	2	2	9	9	9	6	-	-

**Figure 1.**
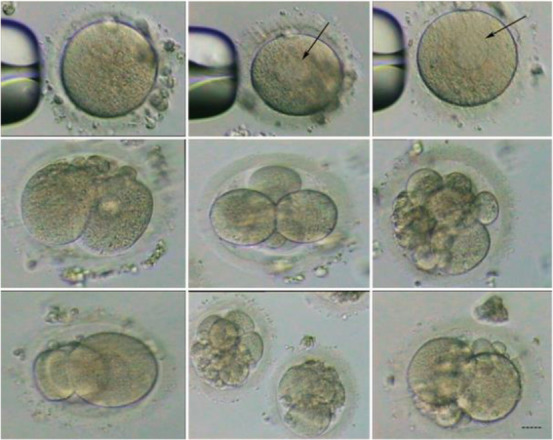
The morphology of oocytes and embryos derived from the infertile woman with an abnormal karyotype. Black arrows show SER cluster in the oocytes. Bar: 20 *µm*

**Figure 2.**
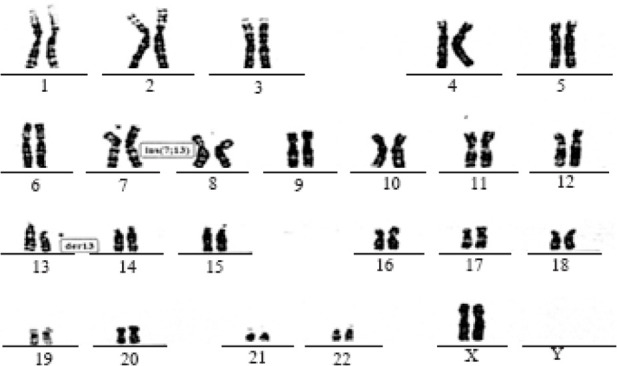
Karyotype analysis in an infertile woman showing a de novo insertion between chromosomes 7 and 13

The oocytes did not demonstrate severe abnormal morphology although one-third of them had smooth endoplasmic reticulum (SER) cluster as described by other studies (6, 7). Next, 16–20 *hr* after sperm injection, oocytes were observed for evaluation of 2PN, but no 2PN was detected (Observations were repeated every 2 *hr*). It was concluded that no fertilization occurred; however, on day 2, embryos were found to be in 2, 3, and 4-cell stages. Few embryos arrived at 5–8 cell stage on day 3–5 but they also were arrested at this stage. The cell divisions in most of the embryos were asymmetric and asynchronous (Figure 1). The results were similar in both ICSI cycles. Finally, the couple referred to the genetic center for karyotyping. While the husband showed a normal male karyotype, analysis in wife revealed an abnormal female karyotype with two rearrangements: 1) chromosome 13 with an interstitial deletion between bands q14.2 and q21.1 and 2) a derivative chromosome 7 containing this segment of chromosome 7 as an insertion onto short arm at the p14 position. The parents of this woman were karyotyped and were found to have a normal chromosome complement. Therefore, this chromosome abnormality is de novo (Figure 2). A written consent form was taken from the couple for publishing the data.

## Discussion

To the best of our knowledge, this is the first report of insertion 46 XX, ins(7:13)(p14; q14.2 q21.1) which is associated with the embryo development arrest following assisted reproductive technique.

The embryo genome is produced by the reprogramming of maternal and paternal chromosomes during the cleavage stage, which is known as embryonic genome activation (EGA). Generally, it occurs at 4–8-cell stage in human. If EGA does not occur at this stage, the preimplantation embryo development is arrested due to lack of normal cellular function in blastomeres (8, 9). There is some instability in chromosome number and structure during the human cleavage stage that is necessary for normal embryo development; however, it also can cause some abnormal conditions such as genetic diseases (10). Among all structural chromosomal abnormalities, insertions (A specific type of translocation) are rare rearrangements that occur following two breaks in the first chromosome and reinserted into an interstitial region of the second chromosome. Insertions have more reproductive risks compared to other arrangements. It is estimated that about one-third of carriers have a child with an abnormal chromosome (11, 13). Some cases of carriers with insertions and their reproductive outcomes are reported by Van Hemel and Eussen. Insertions between chromosomes of (5;11)(p14; q14q24), (11;13)(q14q122) (q21.32q31.2), (18;5)(q21.3; p13.1p14), (18;12) (p11.3; q13q15), and (5;10)(q15; q26.3q25.2) are associated with birth defects and spontaneous abortion (13).

It should be noted that the carriers of structural chromosomal abnormalities have a low chance to generate normal or balanced gametes. The productive outcomes of these gametes depend on the breakpoint positions, the segregation patterns, and the sex of the carrier. Such a low chance is due to abnormal segregation of chromosomes during meiosis (14). Indeed, although the balanced carriers seem to be healthy people, they suffer from reproductive problems due to unbalanced insertions in their embryos (13). In our case, the patient was a healthy and beautiful woman with no appearance of any disease and abnormality. In further evaluations, she displayed de novo chromosomal insertion, 46 XX, ins(7:13)(p14; q14.2q21.1). All examined cells derived from the woman showed similar karyotype, indicating the non-mosaicism in the patient. Considering the normal karyotype of his parents, chromosomal rearrangement is de novo. On the other hand, since the husband has a healthy child from the first marriage, this de novo insertion can justify infertility, history of abortion, and repeated failure of IUI and IVF cycles in the woman.

According to the literature review, chromosomal rearrangements are prevalent in cases of infertility, repeated implantation failure, and recurrent abortions. Indeed, the frequency of chromosomal abnormalities among patients with a history of implantation failure and recurrent abortion is reported to be 2.5% and 4.7%, respectively. In addition, cytogenetic analysis in men with non-obstructive azoospermia (NOA) and severe oligoasthenoteratozoospermia (OAT) showed 7.7% Y-chromosome microdeletions. A few men also showed both chromosomal abnormalities and Y-chromosome microdeletions (15, 16). It is reported that balanced translocations are related to repeated implantation failure while the presence of unbalanced translocations in gametes may disturb preimplantation embryo development (16). Currently, preimplantation genetic diagnosis (PGD) can be used in chromosomal rearrangement carriers to identify their normal and abnormal embryos before transfer (17). During PGD, biopsied blastomeres or trophectoderm from dividing embryos are analyzed genetically and then normal embryos are transferred (18). In our case, performing PGD was not possible due to the embryo development arrest at 2–8 cell stage. Therefore, the couple was consulted to use egg donation programs.

## Conclusion

It is concluded that 46 XX, ins(7:13)(p14; q14.2q21.1) is a novel de novo chromosomal insertion related to the female infertility and embryo development arrest following assisted reproductive technique.
